# Co-Design, Development, and Evaluation of a Mobile Solution to Improve Medication Adherence in Cancer: Design Science Research Approach

**DOI:** 10.2196/46979

**Published:** 2024-04-03

**Authors:** Thu Ha Dang, Nilmini Wickramasinghe, Abdur Rahim Mohammad Forkan, Prem Prakash Jayaraman, Kate Burbury, Clare O’Callaghan, Ashley Whitechurch, Penelope Schofield

**Affiliations:** 1 Department of Psychological Sciences School of Health Sciences Swinburne University of Technology Melbourne Australia; 2 Digital Cancer Care Innovation Department of Health Services Research Peter MacCallum Cancer Centre Melbourne Australia; 3 Digital Health Cooperative Research Centre Sydney Australia; 4 Department of Health and Bio Statistics School of Health Sciences Swinburne University of Technology Melbourne Australia; 5 Epworth Healthcare Melbourne Australia; 6 Optus Chair Digital Health La Trobe University Melbourne Australia; 7 Iverson Health Innovation Research Institute Swinburne University of Technology Melbourne Australia; 8 Digital Innovation Lab, Department of Computer Science and Software Engineering School Software and Electrical Engineering Swinburne University of Technology Hawthorn Australia; 9 Factory of the Future and Digital Innovation Lab School of Science, Computing and Engineering Technologies Swinburne University of Technology Melbourne Australia; 10 Digital and Healthcare Innovation Peter McCallum Cancer Centre Melbourne Australia; 11 Sir Peter MacCallum Department of Oncology The University of Melbourne Melbourne Australia; 12 Caritas Christi and Psychosocial Cancer Care St Vincent's Hospital Melbourne Australia; 13 Department of Medicine St Vincent's Hospital The University of Melbourne Melbourne Australia; 14 Department of Clinical Haematology Peter MacCallum Cancer Centre & Royal Melbourne Hospital Melbourne Australia

**Keywords:** cancer, behavioral science, design science research, digital, medication adherence, mobile solution, Safety and Adherence to Medication and Self-Care Advice in Oncology, SAMSON, mobile phone

## Abstract

**Background:**

Medication nonadherence negatively impacts the health outcomes of people with cancer as well as health care costs. Digital technologies present opportunities to address this health issue. However, there is limited evidence on how to develop digital interventions that meet the needs of people with cancer, are perceived as useful, and are potentially effective in improving medication adherence.

**Objective:**

The objective of this study was to co-design, develop, and preliminarily evaluate an innovative mobile health solution called Safety and Adherence to Medication and Self-Care Advice in Oncology (SAMSON) to improve medication adherence among people with cancer.

**Methods:**

Using the 4 cycles and 6 processes of design science research methodology, we co-designed and developed a medication adherence solution for people with cancer. First, we conducted a literature review on medication adherence in cancer and a systematic review of current interventions to address this issue. Behavioral science research was used to conceptualize the design features of SAMSON. Second, we conducted 2 design phases: prototype design and final feature design. Last, we conducted a mixed methods study on patients with hematological cancer over 6 weeks to evaluate the mobile solution.

**Results:**

The developed mobile solution, consisting of a mobile app, a web portal, and a cloud-based database, includes 5 modules: medication reminder and acknowledgment, symptom assessment and management, reinforcement, patient profile, and reporting. The quantitative study (n=30) showed that SAMSON was easy to use (21/27, 78%). The app was engaging (18/27, 67%), informative, increased user interactions, and well organized (19/27, 70%). Most of the participants (21/27, 78%) commented that SAMSON’s activities could help to improve their adherence to cancer treatments, and more than half of them (17/27, 63%) would recommend the app to their peers. The qualitative study (n=25) revealed that SAMSON was perceived as helpful in terms of reminding, supporting, and informing patients. Possible barriers to using SAMSON include the app glitches and users’ technical inexperience. Further needs to refine the solution were also identified. Technical improvements and design enhancements will be incorporated into the subsequent iteration.

**Conclusions:**

This study demonstrates the successful application of behavioral science research and design science research methodology to design and develop a mobile solution for patients with cancer to be more adherent. The study also highlights the importance of applying rigorous methodologies in developing effective and patient-centered digital intervention solutions.

## Introduction

### Background

Optimal adherence to medication is increasingly one of the top priorities in oncology care [1-3]. Medication adherence (MA) is “the extent to which patients take their medication as recommended by their health care provider” [4]. Despite this importance, the MA rate is very low: only 14% for some cancer regimens [3,5,6]. Poor MA negatively impacts the health outcomes of the patient [3,7-9] and increases pressure on health services and health care fiscal restraints [9,10].

MA is a complex and multifaceted phenomenon that can be influenced by 5 interacting dimensions: socioeconomic and health system factors as well as condition-, therapy-, and patient-related factors [11]. Patient-related factors are the most important [12] because adherence interventions may potentially make the most impact on these factors without necessarily having systemic solutions [11]. Multicomponent interventions that involve collective adherence strategies and are tailored to patients are likely more effective than single-strategy interventions in addressing these factors and improving adherence to oral anticancer medicines [13]. Technology can help to deliver multicomponent interventions more effectively and efficiently [13-15] without requiring too many extra resources, which are already scarce, from the health system [16].

With the rapidly evolving nature and increased uptake of information and communications technologies in the last 20 years [14,17], mobile phone–based interventions have been widely used to address the problem of medication nonadherence, specifically in cancer [18,19]. Literature reviews showed the potential of using technologies such as mobile solutions in promoting MA by providing patients rapid, continuous, and easy access to educational resources and symptom or side effect self-management strategies as well as facilitating direct patient-provider communication [11,15,17]. However, there is very limited evidence on how to develop mobile solutions that meet the needs of people with cancer, are perceived as useful, and are potentially effective in improving MA [1,13,19,20].

### Research Context

In our previous research, we developed REMIND, which is a mobile health system to increase adherence to oral medication in people with chronic myeloid leukemia (CML) [21]. It comprises daily SMS text messages to provide drug reminders and symptom self-care advice, as well as nurse telephone consultations to promote adherence [21]. The development of REMIND was guided by the framework for the development of complex interventions [22]. To understand patients’ experiences of CML and identify their possible facilitators and barriers to adherence, a prior qualitative study was conducted [23]. To increase the acceptability of the intervention, stakeholders (eg, consumers and oncology professionals), were involved in reviewing iterative REMIND revisions and resource manuals [21]. The intervention content and delivery mechanisms were based on theories and available evidence [21].

Findings from the REMIND pilot study [21] showed that most patients reported episodes of nonadherence during the study period. Some reasons for their nonadherence were intentional [24], such as to reduce dose-dependent side effects. Some patients reported unintentional nonadherence [25] due to forgetfulness and miscommunication with health care providers [23]. Health care professionals (HCPs) had challenges in accurately assessing patients’ adherence status and identifying causes of nonadherence [23]. Users found REMIND generally acceptable to use and appreciated its benefits in establishing medication routines, resolution of symptom uncertainty, increased awareness of self-care, and informed decision-making [21].

REMIND had limitations. First, using a design framework specifically for digital interventions is crucial; yet, this was missing in the REMIND system’s development. Second, although stakeholder involvement was reported in the intervention’s development process, a genuine co-design process, defined as “meaningful end-user engagement in research design and includes instances of engagement that occur across all stages of the research process and range in intensity from relatively passive to highly active and involved” [26], was not adopted. Third and last, patients reported some functional errors and expressed their need for an advanced iteration with improved functionality and presentation as well as a more user-friendly application [21].

Given the importance of the medication nonadherence problem that has not been well addressed and the gap in literature on how to develop acceptable, useful, and potentially effective digital interventions to solve the problem, as well as the need to resolve the identified limitations of REMIND, we combined design science research methodology (DSRM) and co-design to develop its new version, named Safety and Adherence to Medication and Self-Care Advice in Oncology (SAMSON) mobile health solution.

### DSRM Cycles and Stages

Over the last couple of decades, design science research (DSR) [27] has been one of the main paradigms characterizing most information system research that aims to design and implement innovative technologies [28,29] through 3 design cycles: rigor, design, and relevance [30]. In 2007, Peffers et al [31] presented 6 process stages of the DSRM: problem identification and motivation, definition of the objective of the solution, design and development, demonstration, evaluation, and communication. Later, Drechsler and Hevner [32] extended the original DSRM with a fourth cycle (change and impact) to capture the dynamic nature of information system artifact design for volatile environments. Furthermore, the DSRM has been used in different health care contexts [29,33,34], demonstrating its importance in developing patient-centered digital health solutions. We adapted these 4-cycle and 6-process DSRM models to direct the steps required for the design and development of the SAMSON mobile health solution (hereinafter SAMSON) to improve MA in cancer. We present SAMSON and describe in detail the process of applying DSRM to design and develop it to answer the research question “How can DSRM be applied to enhance the initial mobile health system to provide a better user experience to improve MA to oral anticancer agents in adults with cancer?” Our study aimed to co-design, develop, and preliminarily evaluate SAMSON.

## Methods

### Overview

**Figure 1:**
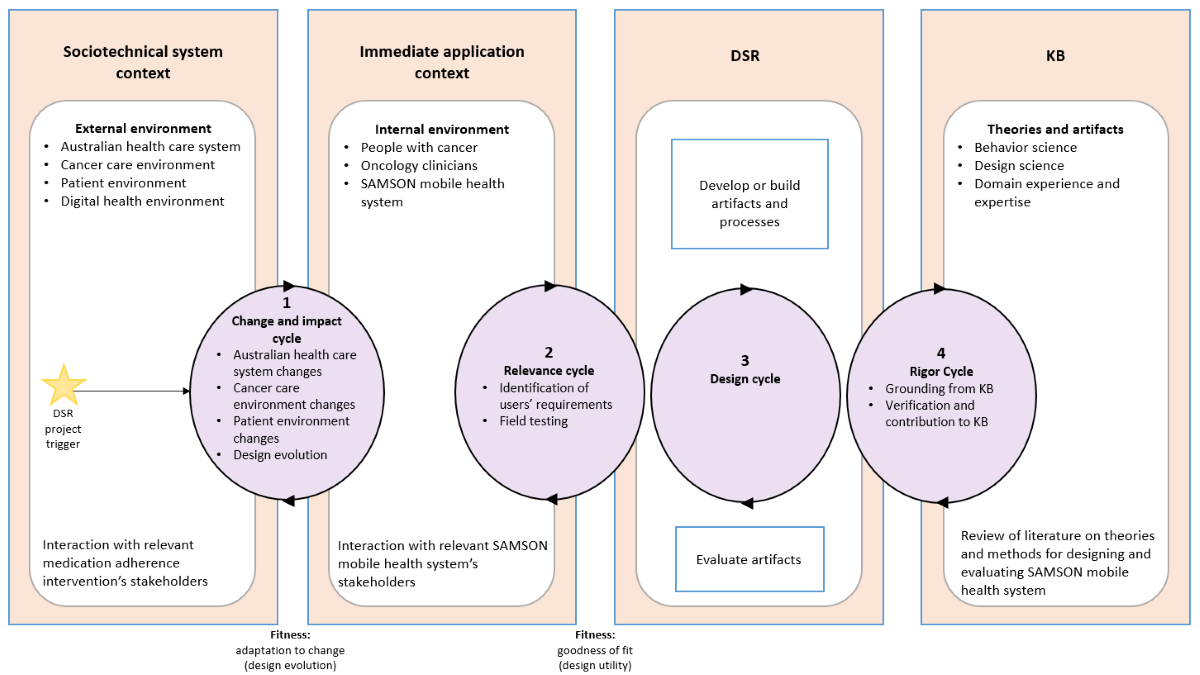
The adapted 4-cycle design science research methodology of the Safety and Adherence to Medication and Self-Care Advice in Oncology (SAMSON) mobile health solution. DSR: design science research; KB: knowledge base.

In this section, we explain how the 4 cycles and 6 processes of DSRM were adapted to design and develop SAMSON. Figure 1 presents the 4 DSRM cycles used to design and develop SAMSON. Table 1 illustrates how the 6-process DSRM models were applied in this study.

**Table 1:** Adapted 6-process design science research methodology (DSRM) applied to design and develop the Safety and Adherence to Medication and Self-Care Advice in Oncology (SAMSON) mobile health solution.

DSRM stages	Interaction of DSRM cycles and stages	Approaches
Stage 1: problem identification and motivation	Cycle 1: change and impact impacts on stage 1	Review literature on MA^a^ problems in cancer Review literature on current MA interventions and their effect Identify problems in the current design Define a set of requirements in the new design
Stage 2: definition of the objective of the solution	Cycle 1: change and impact and cycle 2: relevance impact on stage 2	Review literature on BSR^b^ Adapt BSR principles in design
Stage 3: design and development	Cycle 2: relevance, cycle 3: design, and cycle 4: rigor impact on stage 3	Conceptualize design requirements and features
Stage 4: demonstration	Cycle 4: rigor impact on stage 4	Test the design and acquire feedback from the design’s users
Stage 5: evaluation	Cycle 4: rigor impact on stage 5 Stage 5 impact on cycle 3: design and cycle 4: rigor	Evaluate the acceptability, usability, and potential effect of the intervention
Stage 6: communication	Stage 6 impact on cycle 3: design	Report and publish the evaluation results

^a^MA: medication adherence.

^b^BSR: behavioral science research.

### DSRM Cycles

The change and impact cycle [32] ensures that SAMSON (internal environment) would fit for purpose in the context of the Australian health care system, cancer care, digital health, and patient environments (external environment). The internal environment here includes the designed mobile solution and the users (patients and oncology clinicians). This was defined through the process of problem identification and motivation (DSRM stage 1).

The relevance cycle links the key identified requirements of the users, including the users’ needs from REMIND’s pilot test, and the problems that they are facing in their environments. This was done through a range of discussions with SAMSON’s stakeholders and was demonstrated in DSRM stage 2 (definition of the objective of the solution) and impacted to stage 3 (design and development).

The co-design cycle (phase 1 and 2) consists of smaller cycles or phases (interacting iterative processes), including constructing the artifact, evaluating it, and using evaluation feedback to further refine it until a satisfactory design is achieved [27]. This cycle is the center of the research project because it is directed by the relevance cycle and the rigor cycle [33]. However, this is not a 1-way process because the results of the co-design cycle will then become a part of the relevance cycle. This cycle was performed in DSRM stage 3.

The rigor cycle links design science activities and grounded knowledge bases, such as the scientific theories, experience, and expertise that inform the DSRM project [33]. The scientific theories applied in this study include the health belief model (HBM) [35], self-determination theory (SDT) [36], and behavioral learning perspective (BLP) [11]. The impact of these knowledge bases on the SAMSON was demonstrated in DSRM stages 3 (design and development), 4 (demonstration), and 5 (evaluation). In parallel, the design and use of the SAMSON provide new knowledge (eg, the effect of this solution in terms of promoting adherence among people with cancer) to the external environment (Australian health care and cancer care context) in which the mobile solution is embedded. This process was rigorously validated in stage 5.

### DSRM Processes

**Figure 2:**
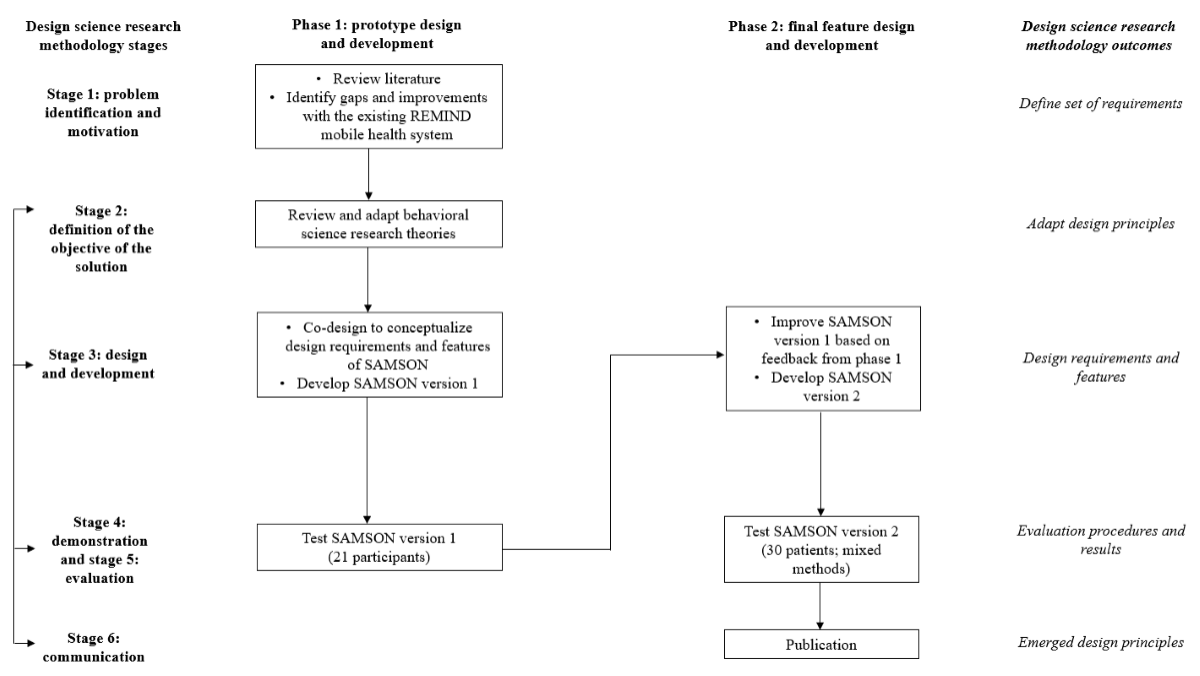
Design and development phases of the Safety and Adherence to Medication and Self-Care Advice in Oncology (SAMSON) mobile solution.

The SAMSON design comprises two phases: (1) prototype design and development and (2) final feature design and development (Figure 2).

#### Phase 1: Prototype Design and Development

The first phase (prototype design and development) started by defining a specific research problem (stage 1). The research problem focuses on MA problems in cancer, barriers to MA, and current MA interventions and their effects. To establish problem awareness, a literature review on MA in cancer and a systematic review of current MA interventions in cancer were conducted by THD and the research team (ARMF, NW, PPJ, and PS) to define MA barriers and facilitators as well as the characteristics of effective MA interventions [13]. Besides, feedback from HCPs and patients regarding REMIND was examined thoroughly by THD and PS to define necessary changes in the next iteration (SAMSON). Guided by the problem awareness, behavioral science research (BSR) was used by THD and PS to conceptualize preliminary design requirements (stage 2). Subsequently, the design requirements were translated into design features for the SAMSON prototype (SAMSON version 1), with ARMF leveraging the available features of the REMIND system in consultation with HD and the research team (stage 3). A test was then conducted on a convenient community sample to investigate whether the prototype works, examine its features, and propose more design requirements that may be helpful for patients (stages 4 and 5). Purposive and snowball sampling [37] were used to recruit participants to test SAMSON version 1. The convenient community sample included project team members, HCPs, and people in the community.

#### Phase 2: Final Feature Design and Development

Feedback from the testing of SAMSON version 1 initiated the second phase (final feature design and development; stage 3). In this phase, the problems detected in phase 1 were fixed. On the basis of feedback from participants in the SAMSON version 1 testing, THD and the research team returned to the literature and consulted the BSR to address the participants’ suggestions and develop the final designed features in SAMSON version 2. A preliminary evaluation of SAMSON version 2 was conducted among people with cancer (stages 4 and 5). Details of the preliminary evaluation study (hereinafter SAMSON evaluation) are presented in the following subsections. The results of the design and development of the SAMSON will be presented in publications (stage 6).

### SAMSON Evaluation Methods

#### Study Design and Setting

This is a study with an explanatory sequential mixed methods design: a quantitative survey was conducted first, followed by qualitative interviews [38]. The quantitative study was conducted using a purpose-built questionnaire. The qualitative component consisted of in-depth interviews with a subset of patient participants. The mixed methods study design was applied to use the qualitative interviews to *explore and make sense* of the quantitative findings [38].

#### Participants

Purposive sampling [39] was used to select outpatient patients from the hematology department at Peter MacCallum Cancer Centre in Melbourne, Victoria, Australia. To be eligible, participants were required to be adults (aged ≥18 years); have an established diagnosis of chronic lymphocytic leukemia, CML, essential thrombocythemia, malignant neoplasm, myelofibrosis, myeloproliferative neoplasms, or polycythemia rubra vera; be taking or commencing an oral anticancer medication; and have smartphone and internet access. Before participating in the study, the study staff helped participants to install the SAMSON app on their mobile phone and briefed them on how to use it. They also received the SAMSON app user manual with detailed information, including app introduction, features, how to install and navigate, and common issues and how to solve them.

#### Intervention

The SAMSON has two elements: (1) a smartphone-based app to remotely prompt MA, monitor the patient’s side effects, and provide self-care advice; and (2) a web-based application to program the patient’s daily drug reminders and side effect surveys, and provide relevant drug information. In this evaluation study, patients were asked to trial the SAMSON smartphone app (the first element). The SAMSON web page (the second element) was used to populate daily drug reminders, weekly side effect surveys, and relevant patient information. Data collected on patients’ self-reported MA and side effects were uploaded and archived on the SAMSON web page.

#### Measures

Patients used the SAMSON app for at least 6 weeks. Subsequently, they were asked to complete the questionnaire via a Qualtrics (Qualtrics International Inc) link [40] that they received in an email from a researcher. The purpose-built questionnaire was adapted from the Evaluation Tool for Mobile and Web-Based eHealth Interventions (Enlight) [41]. The items in the questionnaire were language adapted for respondents without a background in IT and health. Next, face validity testing [42] was applied to achieve a consensus on the adapted Enlight questions. Finally, usability testing following the think-aloud protocol [43] was conducted on 2 consumers to finalize the questionnaire for use.

The questionnaire assesses the quality of the SAMSON app on 6 main constructs or dimensions: usability, visual design, user engagement, content, therapeutic persuasiveness, and general evaluation. Each dimension had between 3 and 6 items, for a total of 25 items. The stem of the item was presented as a statement (eg, “Overall, I found the mobile app was easy to use”), and the response scale was a 5-point Likert scale (1=*strongly disagree*, 2=*disagree*, 3=*neither*, 4=*agree*, and 5=*strongly agree*; Multimedia Appendix 1).

#### Qualitative Interview

All interviews were conducted either face-to-face or via web-based videoconference platform (Zoom; Zoom Video Communications, Inc) by THD, using a semistructured interview guide [39] (Multimedia Appendix 2). Each interview lasted between 45 and 90 minutes and was audio recorded.

#### Data Analysis

##### Quantitative Data

Descriptive statistics were used to analyze the quantitative data. SPSS (version 28.0; IBM Corp) [44] was used.

##### Qualitative Data

Each interview was transcribed verbatim [39]. NVivo 12 qualitative data management software (Lumivero) [45,46] was used. Qualitative data were analyzed thematically using a comparative, iterative, and predominantly inductive process, informed by grounded theory [47,48]. Thematic analysis has been used widely in information system research for different purposes, such as to understand phenomena related to information systems [49] or to evaluate the effectiveness of IT artifacts [50]. A qualitative interrating process was also conducted [51]. First, THD completed coding all interview records. Next, CO reviewed all interviews as well as THD’s codes and agreed or disagreed with each code and also suggested additional codes. Subsequently, both researchers discussed the codes until they reached agreement. Codes were then collated into subcategories (labels for comparable code groups), categories (labels for comparable subcategory groups), and themes (labels for comparable category groups). THD led category and thematic development, which was followed by a review of the categories and themes by CO. All disagreements were also discussed, and adjustments were made until consensus was reached. Both authors reviewed the data to ensure that the themes worked in relation to the entire data set and to generate a thematic map of the analysis. The researchers’ interrating process helped to strengthen the credibility and trustworthiness of this study [52,53].

### Ethical Considerations

The SAMSON evaluation was approved by the human research ethics committees of Peter MacCallum Cancer Centre (HREC/74134/PMCC) and Swinburne University of Technology (20215811-8152). Written consent was obtained from all participants. Throughout the comprehensive consent process, participants were informed that their participation in this research was voluntary and that they were free to withdraw at any stage if they wished to do so. In addition, they were informed that their data would be deidentified for analysis and publication. Participants did not receive any compensation from the research team.

## Results

The results are presented in the sequence of DSRM stages as shown in Figure 2: review literature, review and adapt BSR, co-design and test SAMSON version 1 (design cycle 1), develop SAMSON version 2, and SAMSON evaluation (design cycle 2).

### Review Literature

The literature review of most recent research on MA in cancer showed that the problem of medication nonadherence in cancer is still persistent [5,15]. The results of the systematic review of intervention solutions to enhance adherence to oral anticancer medicines in adults [13] were in line with those of earlier reviews of the same topic [54,55]: multidimensional interventions that use collective strategies (educational, reminder, cognitive, behavioral, and affective) to promote adherence were potentially more effective than single-strategy interventions. This could be explained because MA is a complex and multifaceted phenomenon determined by 5 dimensions—socioeconomic and health system factors as well as condition-, therapy-, and patient-related factors—that require different strategies to address [11,13]. The review also suggested that a combination of cognitive and behavioral theories may better explain the diverse barriers and facilitators to MA and provide stronger direction to formulate interventions [13].

### Review and Adapt BSR

Guided by the problem awareness from the literature review and REMIND studies, we conducted a review of BSR to select cognitive and behavioral theories that can potentially address MA barriers and promote MA facilitators via the SAMSON. The HBM [35], SDT [36], and BLP [11] were chosen to govern the design requirements of the SAMSON mobile solution [56]. According to the HBM, people will take health actions (eg, adherence) if they have 4 basic conditional beliefs or perceptions regarding the disease, the effect of the disease on people’s lives, the action to respond to the disease, and the conviction that the benefit of action will outweigh the barriers [57]. According to the SDT, motivation (intrinsic and extrinsic) is crucial to successful behavior change [36]. The behavioral theory emphasizes the role of positive and negative reinforcements in controlling people’s behaviors that are immediately relevant to adherence [11].

### Co-Design and Test SAMSON Version 1

**Figure 3:**
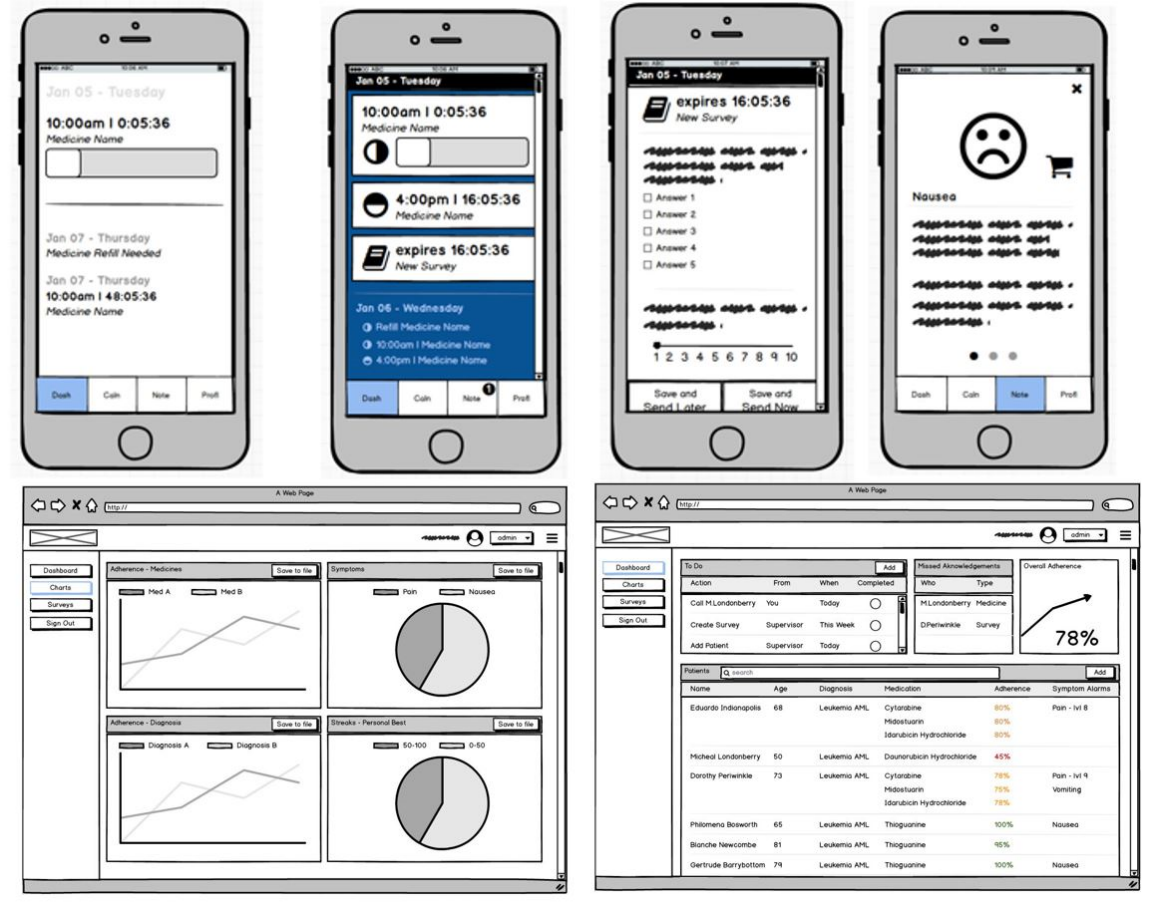
Conceptualization of Safety and Adherence to Medication and Self-Care Advice in Oncology (SAMSON) design requirements using wireframes.

Using knowledge gained from the literature and core theories, as well as users’ feedback on REMIND’s limitations, preliminary design requirements were conceptualized. The outcome of such conceptualization is presented in Figure 3.

During the SAMSON design stage, we focused on the following target behaviors: knowledge, reinforcement, intentions, emotion, social influences, beliefs about capabilities, behavioral regulation, memory, and attention. These behaviors were originated from key barriers to MA, considered most feasible to influence, and expected to contribute most to the improvement of adherence. On the basis of behavioral analysis of these behaviors, potential behavior change techniques (BCTs) from the HBM, SDT, and BLP as well as intervention functions were selected for the SAMSON app; for example, the *prompts* or *cues to action* technique from the HBM [58] was applied for the *medication reminder* feature. *Problem-solving* and *self-monitoring* techniques from the HBM and SDT [36] were applied for the *symptoms assessment and management* module of the app. The *feedback on behavior* technique from the BLP [11] was applied for the *reinforcements* module. Textbox 1 shows the conceptual model picturing this process using BSR, including the selection of final BCTs and the app’s features.

**Conceptualizing Safety and Adherence to Medication and Self-Care Advice in Oncology (SAMSON) design requirements and features using behavioral science research.**

**Requirements and features**

1. Medication adherence barriers
  Drugs’ side effects
  Lack of medication knowledge
  Lack of motivation
  Lack of health care professional (HCP) support to manage side effects
  Poor patient-HCP communication
  Lack of self-efficacy
  Forgetfulness
2. What needs to change
  Knowledge
  Reinforcement
  Intentions
  Emotion
  Social influences
  Beliefs about capabilities
  Behavioral regulation
  Memory and attention
3. Behavior change techniques from the theories
  Information about side effects and medicines
  Feedback on behavior
  Social support
  Problem-solving
  Self-monitoring behavior
  Prompts
  Habit formation
4. App features
  Side effects section
  Medication information section
  HCPs’ contacts
  HCP connections
  Side effects self-management section
  Drug reminders
  Reinforcements

The aforementioned step is followed by a translation into design features for prototype implementation. The features were designed to provide a solution within 1 IT artifact, which is called the SAMSON mobile solution, including a mobile app, a web portal, and a cloud-based database for storing patient-specific information. The mobile app is available for patients to use, while the web portal is available for both patients and their care team. The SAMSON included 5 different modules with some key requirements (Textbox 2).

**The 5 different modules of the Safety and Adherence to Medication and Self-Care Advice in Oncology (SAMSON).**

**Medication reminder and acknowledgment**

- The app should support and display multiple medications and send a reminder per medication. The reminder can address the medication adherence (MA) barrier of forgetfulness. Patients need to tap each reminder for an acknowledgment.

**Symptom assessment and self-care management**

- A list of available side effects and symptoms and self-care management in the mobile app is displayed for the medication that the patient is taking. Patients should be able to complete a symptom assessment survey through the app that will be distributed to patients using app reminders. They should be able to view information on how to manage their symptoms (if minor) and when they need to contact health care professionals (HCPs). This provides patients with medication knowledge as well as support in side effects self-management, both of which are important enablers of MA.

**Reinforcement**

- The app sends a positive reinforcement to the patient at a specific time each week based on the MA profile for that week. Positive reinforcement can help motivate patients’ adherence.

**Patient profile**

- Patients can use the app to view their profile information, such as their basic personal and clinical information, emergency contact and care team contact details, and medication information (both general and important). This information can address the MA barriers of lacking or misunderstanding medication information and poor patient-HCP communication.

**Reporting**

- Analytical reports of patients’ adherence status and their symptoms are available on the web portal for HCPs and patients in real-time. HCPs should be able to use these data in monitoring patients and providing them in-time and tailored support to manage side effects as well as to overcome MA barriers.

### Test SAMSON Version 1

SAMSON version 1 was tested by 21 participants from a convenient community sample, which included project team members, HCPs, and people in the community. We sought participants’ feedback on issues regarding the expected features and functionalities of the prototype and its visual design. Overall, participants reported some functional errors, such as misdelivered medication reminders and data entry failures in some fields both in the smartphone app and on the web page. They also asked for new visual design requirements to meet users’ needs (details are presented in Multimedia Appendix 3).

### Develop SAMSON Version 2

#### Overview

In phase 2, consumers’ feedback from design cycle 1 was reviewed by the project team. We grouped them based on the artifact’s functions and priority in terms of improvement (Multimedia Appendix 3). The main improvements in SAMSON version 2 are described in terms of priority in the following subsections.

#### Priority 1: Fix Functional Errors

All functional errors were fixed in this stage, including misdelivered medication reminders, app log-in–related issues, slow responsiveness to load app content, editing errors of medication schedules on the website, and functional errors related to data saving and data sorting on the website.

#### Priority 2: Enhance Existing Features and Functions

Textbox 3 presents feature and function enhancements in SAMSON version 2 in comparison to version 1.

**Enhanced features and functions in Safety and Adherence to Medication and Self-Care Advice in Oncology (SAMSON) version 2 in comparison to version 1.**

**Version 1**

1. The medication reminders had no expiry time
2. Medications did not have color attributes
3. Patients could not view their data on the website
4. Patients could not export side effects surveys from the website to their data folder

**Version 2**

1. Setting up an expiry time (6 hours) for the reminders
2. Adding color attributes for medications on the website 
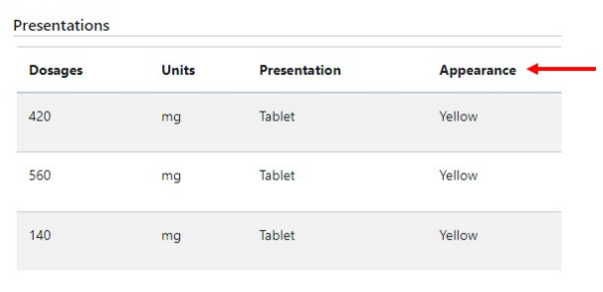
3. Enabling patients to log in to the website to view their own adherence performance, symptom reports, and completed side effects surveys
4. Enabling patients to export the side effects surveys from the website to Excel (Microsoft Corp) spreadsheets

#### Priority 3: Create New Functions

The need expressed by consumers for new functions was discussed by the project team and conceptualized to guide the development of new selected design features in the new iteration of SAMSON. The literature, BSR theories, and BCTs were revisited when necessary to address new suggested requirements. Some selected new functions of SAMSON version 2 in comparison to version 1 are described in Multimedia Appendix 4.

### SAMSON Evaluation

#### Overview

In the SAMSON evaluation, 30 (81%) of the 37 patients who were approached agreed to participate in the study and used SAMSON. None of them withdrew from the study. After 6 weeks, of the 30 participants, 27 (90%) completed the questionnaire, and 25 (83%) participated in the interview (Multimedia Appendix 5). Data retrieved from the SAMSON web page showed that 100% (1890/1890) of the drug reminders were sent on time, and all participants responded to the reminders and viewed the reinforcement messages. Most of the participants (23/30, 77%) reported side effects during the study period.

Participant demographics are described in Table 2. The mean age of the patient participants was 57.6 (SD 12.5) years. Most of the participants (20/27, 74%) were male individuals. The average time that participants had received treatments before the start of the study was 7.2 (SD 6.7) years. Approximately two-thirds of the participants (17/27, 63%) lived in the metropolitan areas of Melbourne. The proportions of participants using iPhones and Android mobile phones were equal.

**Table 2:** Participant demographics and clinical characteristics.

Characteristics			Values
Age (years), mean (SD)			57.6 (12.5)
**Sex (n=27), n (%)**			
	Male	20 (74)	
	Female	7 (26)	
**Country of birth (n=27), n (%)**			
	Australia	14 (52)	
	Croatia	1 (4)	
	India	3 (11)	
	New Zealand	1 (4)	
	Pakistan	1 (4)	
	United Kingdom	2 (7)	
	Not provided or missing	5 (19)	
**English as first language (n=27), n (%)**			
	Yes	25 (93)	
	Not provided or missing	2 (7)	
**Education (highest level completed; n=27), n (%)**			
	No formal schooling or incomplete schooling	1 (4)	
	Primary school	9 (33)	
	Secondary or high school	3 (11)	
	Vocational	6 (22)	
	University	7 (26)	
	Postgraduate diploma or master’s degree or PhD	1 (4)	
**Employment status (n=27), n (%)**			
	Working full-time	8 (30)	
	Working part-time	5 (19)	
	Casual	2 (7)	
	Sick leave (permanent)	2 (7)	
	Unemployed	1 (4)	
	Retired	9 (33)	
**Residence (n=27), n (%)**			
	Metropolitan	17 (63)	
	Rural	10 (37)	
Time since diagnosis (years), mean (SD)			7.2 (6.7)
**Diagnosis (n=30), n (%)**			
	Chronic lymphocytic leukemia	15 (50)	
	Chronic myeloid leukemia	12 (40)	
	Essential thrombocythemia	1 (3)	
	Myeloproliferative neoplasms	1 (3)	
	Polycythemia rubra vera	1 (3)	
**Mobile phone operating system (n=30), n (%)**			
	Android	15 (50)	
	iOS	15 (50)	

#### Quantitative Survey

Participants’ responses to the Enlight questionnaire are presented in Multimedia Appendix 6. Enlight aims to examine individual quality constructs or dimensions, which means it is a suite of scales rather than 1 quality measure; therefore, we did not present the overall scale of the questionnaire.

Usability assesses the ease of learning how to use an app and the ease of using it properly. Overall, of the 27 participants, 21 (78%) rated the app as *easy to use*; only 1 (4%) participant rated it as *difficult*.

Visual design assesses the look and feel of an app. Participants mentioned that the app is attractive (14/27, 52%); well organized (19/27, 70%); and has appropriate font size, buttons, and menus (23/27, 85%). Some of them expressed a need for the app to be redesigned to increase its appeal (3/27, 11%) and encourage engagement (5/27, 19%).

User engagement assesses the extent to which an app’s design attracts people to use it. In general, participants were interested in using the app (18/27, 67%) because it was presented in an interesting way (19/27, 70%), different features were used to increase users’ interactions (19/27, 70%), automated features to respond to the survey were easy to use (21/27, 78%), and the app’s features were personalized to users (23/27, 85%). However, of the 27 participants, 2 (7%) were not interested in using the app at all.

In terms of the content, more than two-thirds of the participants (19/27, 70%) were satisfied with the amount of information and the way it was presented in the app. Nevertheless, 7% (2/27) of the patients thought that information about the app’s purpose was missing. Patients also reported that information about drugs and side effects was presented with gaps, overexplanation, or irrelevance (5/27, 19%).

Therapeutic persuasiveness assesses the extent to which an app is designed to encourage a patient’s MA. High proportions of participants agreed that the app provided activities to improve their adherence (21/27, 78%) and appropriate ongoing feedback (19/27, 70%). However, approximately one-fifth of the participants (6/27, 22%) did not think that completing activities on the app would help them to be more adherent to treatments. Patients thought that the app did not fully disclose information on how it can help them to be more adherent (7/27, 26%) and what they need to do for this (8/27, 30%).

Overall, 18 (67%) of the 27 participants thought that the app was valuable in assisting MA via improving their confidence in complying to treatments (11/27, 41%) and motivating them to do so (15/27, 56%). More than half of the participants (17/27, 63%) would recommend the SAMSON app to other people with cancer.

#### Qualitative Interview

##### Overview

**Figure 4:**
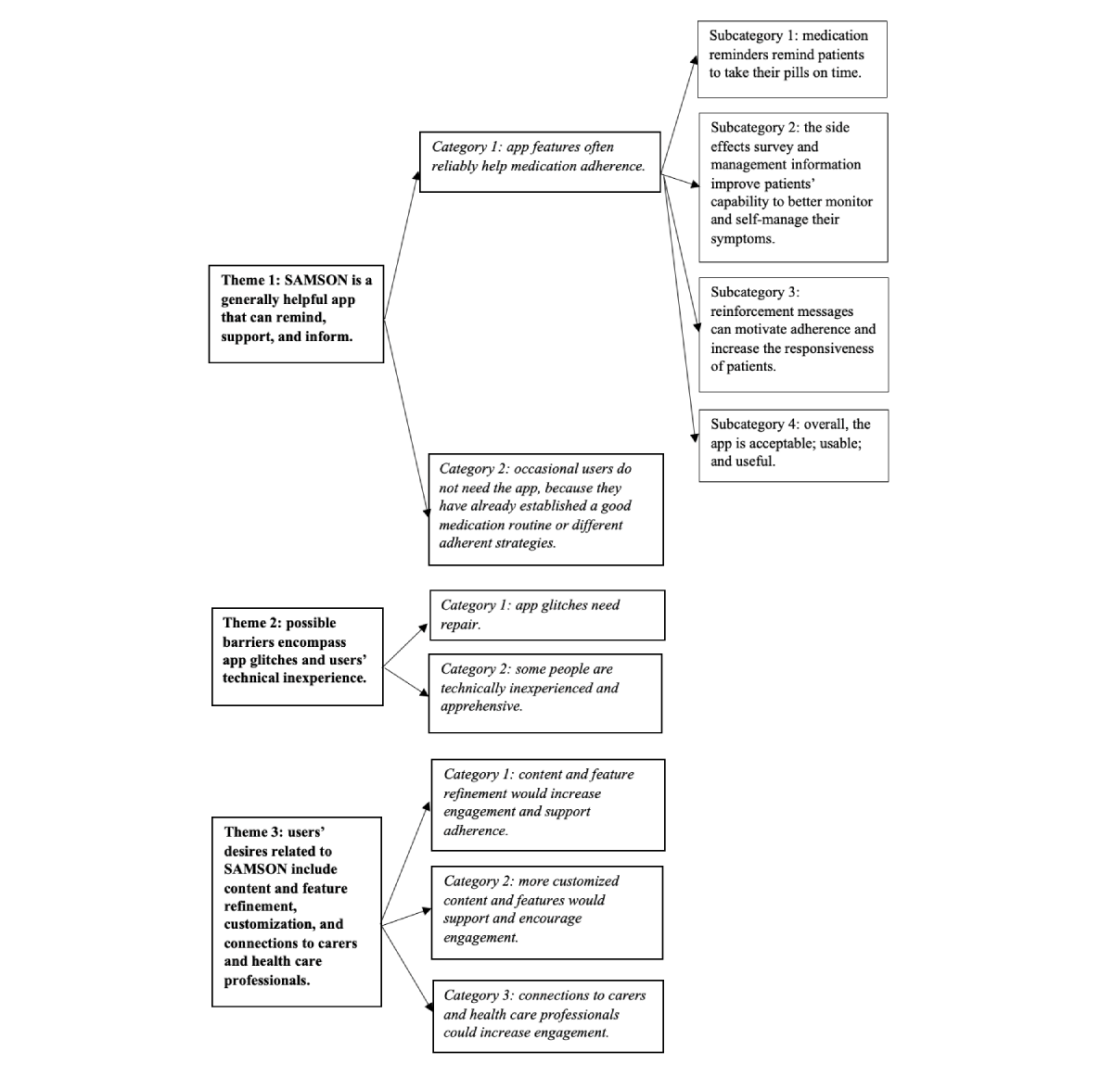
Qualitative interviews: themes, categories, and subcategories mapping. SAMSON: Safety and Adherence to Medication and Self-Care Advice in Oncology.

Three common themes were generated from the interview data: (1) SAMSON is a generally helpful app that can remind, support, and inform; (2) possible barriers encompass app glitches and users’ technical inexperience; and (3) users desire customization, health care connections, and content refinement of SAMSON (Figure 4). A full presentation of themes, categories, and subcategories is presented in Multimedia Appendix 7. Further clarification of the themes is provided in the following subsections.

##### SAMSON Is a Generally Helpful App That Can Remind, Support, and Inform

Participants valued the SAMSON app’s features (eg, medication reminders and side effects information) because they are reliable, both in terms of content and functioning. As patients had to take >1 drug, different drug schedules and a busy life made it easy to forget taking pills on time. However, with the use of SAMSON, patients were reminded to take their medication on time:

[U]sually the reminder comes very close to the time...I do like that.

P6

[T]he medication reminder is very prompt, and I have been, you know, taking my medication regularly, absolutely, I never missed out.

P11

Although patients had access to different sources of information about the drug and its side effects, they found it easy to obtain the appropriate information that they needed in SAMSON:

[W]hen you go into the individual drugs and you’ve got the side effects, information is much easier to access from the app than it is if you go online. Or if you go into the drug information sheet, which is just, you know, overwhelming, it’s difficult. Particularly for somebody without a medical or paramedical background. But I thought the content was really well done. So you know I could find what I needed to know...it was a very good summary.

P13

A participant and their carer trusted the app’s information because it was based on reputable research:

[O]n the Samson app, you can go through and actually know you can trust what’s in there...you guys and [Swinburne University of Technology] have done the research.

P1 and carer

Patients could also benefit from information about drugs and side effects in the app. It supported them in managing unpleasant symptoms as consequences of cancer treatments that can be discouraging:

The side effects part of the screen was helpful to me in in a simple fashion...support for people that are trying to manage their side effects. I thought it was good...excellent.

P14

SAMSON could also encourage MA maintenance via reinforcement messages:

I liked the way it encouraged. You know, I was like, you know, try better this week, you know, do better.

P1

It is encouraging the patient to always use the app and to take the medicine on time.

P12

Overall, most of the participants thought that SAMSON is a helpful digital solution to promote MA:

It does its job, so it’s good...It’s a very neat app in this in the sense there’s no extra stuff. It’s just exactly just what it needs to do. That’s all, so yeah, it’s pretty good.

P7

However, some of the participants mentioned that they did not need the app to comply with the treatment because they either had a well-established drug-taking routine for many years or had another medication reminder strategy:

I’ve got a container with my medications in, as soon as I get them, I write the dates on there, I know exactly, I don’t need my phone, I don’t need an app, I don’t need anything to remind me, I know, it’s a routine that I’ve done for too long...I’ve been on it over 14 years, so for me it’s a daily thing.

P24

Nonetheless, these participants still praised the app as helpful for other patients, especially for those who are new to the treatment and like to use technology:

I can see when someone’s first-time treatment it’d be very very useful.

P9

A newer person coming into their treatment, or a younger person that’s a bit more tech savvy, would probably prefer to use technology as a reminder. And you know that would be very handy.

P10

##### Possible Barriers Encompass App Glitches and Users’ Technical Inexperience

Despite the benefits that SAMSON can bring to patients, it has some functional errors and drawbacks. These could annoy patients and make the app less effective:

I’ve got an Android [mobile phone] and I had to refresh the page many, many, many times.

P14

The notification does come up, but it sits in the background on your phone, so it comes up separate from the app, as a notification. But it just sort of sits in the background.

P30

Besides, as in the case of other advanced technologies, the use of SAMSON could be challenging for some people, especially older adults and people who are not technology savvy. A participant reflected on how others might view the app:

I think an app like that for my father who’s in his 80s, I couldn’t see it being used, he’d see it as a nuisance.

P9

##### Users’ Desires Related to SAMSON Include Content and Feature Refinement, Customization, and Connections to Carers and HCPs

Patients expressed their desire for refinements in the new version of SAMSON to make it more appealing and capable of serving diverse needs of different users:

Maybe you can increase the size or to magnify for people.

P31

[P]ossibly people might find something that gives them their compliance, or you know a color changes, [signifying] you're on track, no you’re not. That may help them.

P7

New features were also suggested to improve SAMSON’s effectiveness for both patients and their clinicians in disease management:

It might be handy on the app somewhere for the person using it to be able to make a note and say, put dates in “I’ve been in hospital” or “I’ve had broken, been in a car accident” or “I’ve got some bruising as a result of a fall” or something.

P10

You probably need to have areas where people can actually add things to it, other than just keep going click click click and then get nothing at the end...it doesn’t really help...a section where you can add additions to it, even say a basic of when there’s a section on gastro and vomiting...did it affect you for a percentage...and then you may be able to assist from that side.

P24

App modifications were also advised to improve patients’ proactiveness in treatment management:

It makes you feel more like you’re in control and that you can I think you’re more likely to use an app if you can customize it to meet your own needs. And, whereas you know if I wanted to change it, and then I had to get in touch with someone to do that. Yeah, it’s just a bit disempowering.

P27

Furthermore, patients emphasized new features to assist carer engagement with the app, which would support their MA, and communication with their HCPs when needing additional support:

Some patients, they don’t have this ability to manage their own medications, even when they have the app, and they need carer or family member to be also involved in the app.

P12

There were times in the past that I might’ve had a side effect, or something had happened, and sometimes it was very hard to contact the nurse that’s linked in with my hematologist. So to have something like that [2-way SMS feature] on the app would be good because you could get almost feedback a lot quicker.

P10

## Discussion

### Overview

This research study co-designs, develops, and evaluates an innovative mobile health solution to improve MA in cancer. Preliminary results demonstrate the successful application of BSR and DSRM to enhance the initial mobile health system and provide a better user experience. The study contributes to theory and practice in many ways.

### Theoretical Contributions

Our study contributes to DSRM theory in 4 different ways. First, we expanded the scope of DSRM by applying it to the design and development of a mobile health solution for MA. Given the current challenges in public health and clinical fields, the potential of using DSRM to improve the effectiveness and efficiencies of health care innovations is enormous [33]. DSRM has been used to design new artifacts in different health care application areas [29,34] (eg, medical devices), but none of them target MA in cancer [13,33]. Hence, this study sheds new light on how DSRM can be applied in this area.

Second, we addressed the knowledge gap on how BSR and DSRM can be integrated to develop engaging and effective behavior change digital health solutions. There is a strong view that design science and behavioral science are 2 distinct research paradigms [27]. Design science is related to the creation of new artifacts, while behavioral science studies behavior in relation to IT use [59]. While behavioral science could be seen as a reactive and retrospective process to explain what already exists, design science is more proactive in its way in terms of creating technological solutions for the future [59]. Nevertheless, these 2 seemingly divergent research paradigms have some similarities. They both emphasize the importance of understanding the health problem before designing a solution and aim to ensure that the designed solutions can effectively engage users [60]. Engagement with mobile health interventions is a precondition for their effectiveness [61]. Behavior change theory and BCTs can assist macroengagement with the behavior changes the mobile health intervention aims to support (eg, MA) [62], while design science approaches, such as user-centered design, is more likely to facilitate microengagement with the mobile health interface (eg, logging in to the app) [60,63]. Therefore, integrating best practices from BSR and DSRM can bring more mutual benefits to design engaging behavior change interventions [60]. Research also showed that digital interventions developed using behavior change theory and BCTs are more likely to be effective than those without [60,62]. However, little is known about how these 2 approaches can be blended throughout the design process of artifacts to ensure that microengagement and macroengagement needs will be met [60]. Here, our study provided more understanding about how this integration can be done.

Third, an explanatory sequential mixed methods design [38] was applied in the evaluation stage of the SAMSON’s DSRM. This type of design is helpful for us to know why the user rated the solution’s quality as *low* or *high* for each criterion and gain further understanding on how we can improve the SAMSON in its next version. Because of the assumption that technical knowledge is needed to complete the questionnaire, we adapted Enlight for a lay audience. Unlike some other assessment tools, Enlight includes some quality constructs associated with intervention outcomes, such as persuasive design, behavior change, or therapeutic alliance [41], which is specifically necessary for a mobile solution, such as SAMSON, to change patients’ behavior toward medical treatments. This tool has been validated for assessing eHealth interventions regardless of delivery mediums and clinical aims [41]. In our study, it was language adapted but requires further validation for a community sample. Measures for evaluating the quality of a designed artifact are often difficult to define and are controversial [33]. Therefore, applying a mixed methods design with an appropriate, reliable, and valid assessment tool in the evaluation of digital interventions (eg, in the case of SAMSON) could be one of the effective ways to address this challenge.

Fourth, we effectively involved stakeholders, including real users, early and throughout the co-design and evaluation processes. We formed a project steering committee that included experts in allied health, app development, computer sciences, digital health, oncology, and psychology, as well as consumer representatives. They were involved very early in the co-design process to guide the review of behavior change literature and the selection of targeted change in nonadherence behavior. The committee was also involved in reviewing problem identification and design motivation, adapting BSR principles, and conceptualizing design requirements for SAMSON. After development, SAMSON was thoroughly tested by reasonable numbers of users (21 consumers tested version 1, and 30 patients tested version 2). Their feedback in the testing was then used to construct new requirements or refine the next version of SAMSON. By recognizing users as experts of their own experience, the proper co-design process can address pitfalls in the design and development of mobile health solutions that might limit adoption and effective use in practice [64-66] by facilitating necessary collaborations of diverse stakeholders [67,68] and leveraging expert insights and best practices [69,70].

Our study also contributes to the literature of interventions to promote MA in cancer. Systematic reviews of MA interventions in adults with cancer showed that there was limited use or poorly reported use of theory [71] and frameworks [20] in the design and development of digital interventions [13]. A high number of MA digital interventions have been proposed, but many of them have low user acceptance [72], and their effectiveness in clinical oncology practice is poorly supported [17,20]. Perhaps poor design is 1 reason for these issues [33]. To the best of our knowledge, SAMSON is one of the very first mobile health solutions to improve MA in cancer that applied rigorous DSRM in the design and development process. The use of DSRM provided various improvements in identifying and addressing requirements as well as evaluating this digital solution. SAMSON was perceived as acceptable, usable, and useful by end users.

### Implications for Practice and Future Research

Broadly, our study’s findings have implications for behavioral science and design science researchers, MA intervention developers, and oncology care providers. These findings provide additional evidence on the use of DSRM in health innovations. They can be used to develop principles for guiding DSRM adaptation and BSR integration in the design and development of mobile health solutions in general as well as those targeting MA. The findings of this work provide insights for oncology care providers to use, while encouraging the use of digital solutions to promote MA and drive health care outcomes. Technologies can enhance measures to improve MA, such as patient education as well as side effect monitoring and reporting, and facilitate effective communication between patients and their care team.

Our respondents indicated their acceptance of the mobile solution and valued its usability and usefulness in supporting their adherence to medication. They also reported some functional errors and the need for some further improvements in the design and features of SAMSON. We will use these findings to refine SAMSON and evaluate its acceptability, usability, and effectiveness in a future randomized controlled trial. On the basis of the feedback of participants, in the trial’s protocol, we will include assessments to help identify those who would benefit from the SAMSON.

### Limitations

This study has limitations. Participants enrolled in the SAMSON preliminary evaluation are from the Peter MacCallum Cancer Centre hematology department, and most of them used only 1 oral anticancer regimen. As Peter MacCallum Cancer Centre is one of the leading oncology hospitals in Australia, in the interview, patients acknowledged that the care service that they received was of high quality. Many were provided medication education before starting treatments and at ongoing follow-up appointments. As a result, their perceptions of MA solutions may not represent those of patients who use multiple anticancer medicines and receive care from low-resource oncology settings. Future research can extend the evaluation of SAMSON to patients with other types of cancer at different levels of oncology care institutions.

### Conclusions

By following the systematic DSRM approach, a patient-centered mobile health solution was developed to meet the needs and preferences of people with cancer and thus highly likely to be used by end users. This extensive report of the intervention development process provides transparent guidance on how to develop patient-centered digital mobile health solutions that will have a high likelihood of uptake.

## Appendix

Multimedia Appendix 1 Evaluating the Safety and Adherence to Medication and Self-Care Advice in Oncology (SAMSON) mobile app questionnaire.

Multimedia Appendix 2 Interview guide.

Multimedia Appendix 3 Feedback from consumers on Safety and Adherence to Medication and Self-Care Advice in Oncology (SAMSON) version 1.

Multimedia Appendix 4 New designed features of Safety and Adherence to Medication and Self-Care Advice in Oncology (SAMSON) version 2 in comparison to version 1.

Multimedia Appendix 5 CONSORT (Consolidated Standards of Reporting Trials) diagram.

Multimedia Appendix 6 Safety and Adherence to Medication and Self-Care Advice in Oncology (SAMSON) participant rating.

Multimedia Appendix 7 Qualitative analysis codebook.

## Abbreviations

BCT: behavior change technique
BLP: behavioral learning perspective
BSR: behavioral science research
CML: chronic myeloid leukemia
DSR: design science research
DSRM: design science research methodology
Enlight: Evaluation Tool for Mobile and Web-Based eHealth Interventions
HBM: health belief model
HCP: health care professional
MA: medication adherence
SAMSON: Safety and Adherence to Medication and Self-Care Advice in Oncology
SDT: self-determination theory

## Data Availability

The data sets generated and analyzed during this study are available from the corresponding author on reasonable request.
