# Supplementary material for: Co-Design, Development, and Evaluation of a Mobile Solution to Improve Medication Adherence in Cancer: Design Science Research Approach
Source: JMIR Cancer. 2024 Apr 3;10:e46979. doi: 10.2196/46979 (PMC11024750; doi:10.2196/46979)
Supplement: Multimedia Appendix 1 [file cancer_v10i1e46979_app1.doc]

**Appendix 1**

**Evaluating the SAMSON Mobile App**

Participant ID number: ___________________________

Today’s date (DD/MM/YYYY): _______/________/__________

SAMSON mobile app version: Beta 2.0

Your smartphone is: 🞎 iPhone 🞎 Android

**ABOUT THIS QUESTIONNAIRE**

You are invited to complete this questionnaire to evaluate the SAMSON mobile app that you have been using. SAMSON is a smartphone-based application to remotely prompt taking medications as prescribed, monitor patient side-effects, and deliver self-care advice. This research project is supported by research funding from (name withheld from review).

Your opinions are crucial for us to refine the SAMSON mobile app to improve medication adherence among people with cancer. When you have completed the questionnaire, please check through it to make sure you have not missed any questions.

**INSTRUCTIONS**

This questionnaire includes 36 questions and divides into two sections: demographics and evaluating the SAMSON mobile app. It should take you around 20 minutes to complete.

Most questions require you to circle the options (numbers) which best reflect your experience, opinion or situation.

***Thank you for taking part in this study. Your participation is greatly valued.***

**If you have any queries while you are filling out the questionnaire, please contact:**

(name withheld from review)

**SECTION A – DEMOGRAPHIC QUESTIONNAIRE**

This section includes 11 questions. In this section of the questionnaire, we are interested in some background information about you. Please either write the answer or circle the option that best describes you or your current situation.

| 1. How old are you? __________ 2. What is your gender?    1. Male    2. Female    3. Non-binary    4. Prefer not to say 3. What is your current postcode?__________ 4. In which country were you born?    1. Australia    2. Other (specify):___________________ 5. What language do you mainly speak at home?    1. English    2. Other (please specify):_____________ 6. What is your current marital status?    1. Never married/Single    2. Married/De Facto    3. Separated/Divorced    4. Widowed 7. With whom are you currently living with? (Please select all that apply).    1. Alone    2. Partner    3. Parent(s)    4. Siblings    5. Child/children    6. Friends    7. Carer    8. Other (specify):   ____________________________ | 1. What is/was your occupation?   _________________________________   1. Which one of the following best describes your current employment situation?    1. Working full-time (*at least* 38 hours per week)    2. Working part-time (*less than* 38 hours per week)    3. Casual    4. Sick leave/leave of absence – temporary    5. Sick leave/leave of absence – permanent    6. Unemployed    7. Retired    8. Home duties    9. Studying    10. Other: ________________________ 2. What is the highest level of formal education that you have *completed*?    1. No formal schooling/incomplete schooling    2. Primary school    3. Secondary/High school    4. Vocational    5. University    6. Postgraduate    7. Other (specify):   _____________________________   1. Thinking about your diagnosis of CLL, CML or MPN, what year were you first diagnosed?   (enter year first diagnosed):_____________ |
| --- | --- |

**SECTION B – MOBILE APP RATING SCALE**

This section includes 25 statements. For each of the statements below, circle the number that most accurately represents your views where: 1 = Strongly Disagree, 2 = Disagree, 3 = Neither, 4 = Agree, and 5 = Strongly Agree.

|  | Strongly Disagree | Disagree | Neither | Agree | Strongly Agree |
| --- | --- | --- | --- | --- | --- |
| 1. I found it easy to use the mobile app at the beginning. | 1 | 2 | 3 | 4 | 5 |
| 1. I found it easy to move from one screen to another and back. | 1 | 2 | 3 | 4 | 5 |
| 1. Overall, I found the mobile app easy to use. | 1 | 2 | 3 | 4 | 5 |
| 1. The mobile app’s presentation is attractive to me. | 1 | 2 | 3 | 4 | 5 |
| 1. The mobile app’s presentation is encouraging me to use it. | 1 | 2 | 3 | 4 | 5 |
| 1. The mobile app’s information is well organised. | 1 | 2 | 3 | 4 | 5 |
| 1. The size of fonts, buttons and menus are appropriate. | 1 | 2 | 3 | 4 | 5 |
| 1. The mobile app’s content is presented in an interesting way. For example, there is a right mix of text and graphics. | 1 | 2 | 3 | 4 | 5 |
| 1. The mobile app uses different features to increase user’s interactions. For example, sending reminders to take medication, text messages, and feedback. | 1 | 2 | 3 | 4 | 5 |
| 1. The mobile app’s automated features to respond to the reminder and the survey are easy to use. For example, sliding, clicking, or swiping to provide responses. | 1 | 2 | 3 | 4 | 5 |
| 1. The mobile app’s features are personalised for me. For example, using my name in reminder messages and surveys. | 1 | 2 | 3 | 4 | 5 |
| 1. In general, the mobile app is interesting to use. | 1 | 2 | 3 | 4 | 5 |

|  | Strongly Disagree | Disagree | Neither | Agree | Strongly Agree |
| --- | --- | --- | --- | --- | --- |
| 1. There is sufficient information about the mobile app’s purpose. | 1 | 2 | 3 | 4 | 5 |
| 1. There is enough information about drugs and side-effects presented without any gaps, over-explanations, or irrelevance. | 1 | 2 | 3 | 4 | 5 |
| 1. Information is presented clearly in the mobile app. | 1 | 2 | 3 | 4 | 5 |
| 1. The mobile app provides explanations about how it can help me to improve my medication adherence. | 1 | 2 | 3 | 4 | 5 |
| 1. It provides explanations about what I need to do to improve my medication adherence. | 1 | 2 | 3 | 4 | 5 |
| 1. It sets up activities for me to improve my medication adherence. For example, responding to reminders to take medications, answering the weekly side-effects survey, and reporting serious side effects. | 1 | 2 | 3 | 4 | 5 |
| 1. I found the mobile app’s activities quite easy to complete. | 1 | 2 | 3 | 4 | 5 |
| 1. I think that completing activities on the mobile app will help me take my medications as prescribed. | 1 | 2 | 3 | 4 | 5 |
| 1. The mobile app provides appropriate ongoing feedback about my medication adherence. | 1 | 2 | 3 | 4 | 5 |
| 1. The mobile app provides features to assist me in improving my medication adherence. | 1 | 2 | 3 | 4 | 5 |
| 1. The mobile app makes me feel confident that I can use it to improve my medication adherence. | 1 | 2 | 3 | 4 | 5 |
| 1. The mobile app motivates me to use it to improve my medication adherence. | 1 | 2 | 3 | 4 | 5 |
| 1. I would recommend the mobile app to other people I know with cancer. | 1 | 2 | 3 | 4 | 5 |
