# Supplementary material for: Co-Design, Development, and Evaluation of a Mobile Solution to Improve Medication Adherence in Cancer: Design Science Research Approach
Source: JMIR Cancer. 2024 Apr 3;10:e46979. doi: 10.2196/46979 (PMC11024750; doi:10.2196/46979)
Supplement: Multimedia Appendix 2 [file cancer_v10i1e46979_app2.doc]

Appendix 2

**A beta-testing of SAMSON mobile health application on oncology patients.**

**INTERVIEW GUIDE**

Participant ID #: _____________________ Time: ____________________

| **INTRODUCTION & SUMMARISED INTERVIEW PROCEDURE** |
| --- |

1. Interviewer (research coordinator) introduces herself.
2. Interviewer explains the purpose of the interview is to explore the needs of oncology patients regarding information and support to adhere to medication treatments and manage issues relating to drug treatments in their homecare setting; and to provide opportunities for patient’s expectations, experience and perceptions of SAMSON.
3. Interviewer explains the interview procedure:

- Total interview time is about 30-40 minutes
- Interview will be recorded, then transcribed for further analysis
- All of the patient’s information will be de-identified after the interview. This means that patient’s name will be replaced by a unique participant code, so that their personal identity is not connected with their data.
- Patient does not have to answer questions if they do not want to.
- Patient can stop the interview anytime if they do not want to participate any longer.

1. Interviewer informs the patient that she will now start the interview and the audio/video tape will now be turned on.

| **NEEDS OF INFORMATION AND CARE SUPPORT** |
| --- |

1. **Treatments**
2. How long have you received oral cancer drug therapy for [*the name of cancer type*]?
3. **Disease and treatment experience**
4. In your opinion, how important is it for you to take your drugs as prescribed for your cancer? Why? Why not?
5. Are there times when you haven’t taken your medication as prescribed? *Can you tell me more about why you sometimes don’t take your* medication?
6. Are there any drugs that you find harder to take as prescribed? Which ones? Why are these harder?
7. *(If the patient adhered well to the treatment)* What strategies helped you most to remember to take your drugs daily?
8. What side-effects of your medication have you experienced?
9. What side-effects may make you reduce your medication dose or stop using your medicaiton?
10. What strategy did you use to limit these side-effects? What source of information did you find these strategies from?
11. **Expectation**
12. What do you think might be most helpful to you to support you to take your medication as prescribed?
13. Many people who have been diagnosed with cancer have questions and concerns about the medication they are prescribed. What questions or concerns relating to your medication would you like to ask?

| **EXPERIENCE, EXPECTATION AND PERCEPTION OF THE MOBILE SOLUTION** |
| --- |

**The mobile app**

1. How was your overall experience with SAMSON mobile app? specifically, about:

- Medication reminder notifications
- Motivational messages that came with the weekly adherence rate
- Side-effects survey
- Ability to report serious side-effects
- Side-effects advice pages

1. What did you like about using the app?
2. What did you dislike about using the app?
3. If you were an app designer, what would you do to improve SAMSON?

| **INTERVIEW ENDING** |
| --- |

The interviewer asks the patient if they have any questions or comments and thanks patient for their time that they have given to the study.
