# Supplementary material for: Co-Design, Development, and Evaluation of a Mobile Solution to Improve Medication Adherence in Cancer: Design Science Research Approach
Source: JMIR Cancer. 2024 Apr 3;10:e46979. doi: 10.2196/46979 (PMC11024750; doi:10.2196/46979)
Supplement: Multimedia Appendix 4 [file cancer_v10i1e46979_app4.docx]

**Multimedia Appendix 4**

New designed features of Safety and Adherence to Medication and Self-Care Advice in Oncology (SAMSON) version 2 in comparison to version 1.

| **Version 1**   1. All patients received the same side effects survey regardless of the drugs they used 2. The side effects survey was created manually on the web page 3. Patients could not see their progress when completing the side effects survey   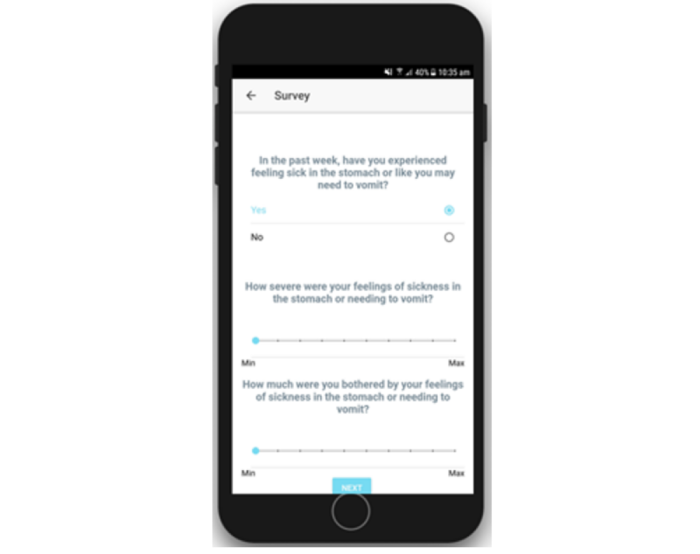   1. There was no search function on the web page 2. It was hard to link the drug’s brand name on the SAMSON web page with a similar drug (with a different brand name) in another database 3. There was no “welcome” page on the SAMSON smartphone app 4. There were no images of side effects 5. The medication reminders and side effects were not grouped together on the home screen   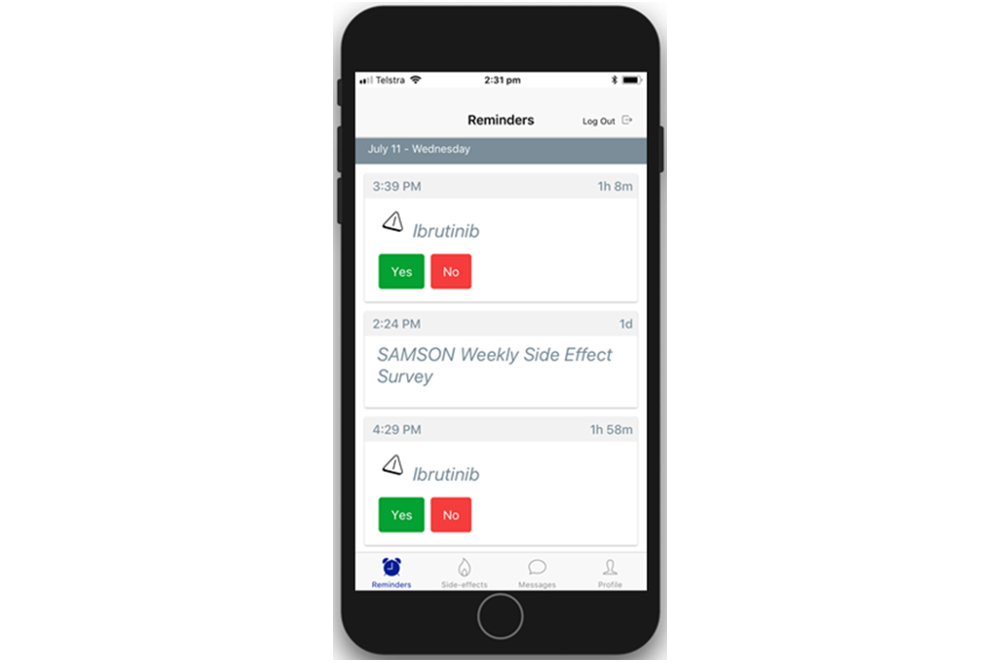  **Version 2**   1. The side effect surveys are tailored to patients’ drugs 2. All side effects surveys are systematically automatically generated by the system, based on the list of common side effects of specific medicines that are imported into the system’s database; of note, before importing to the system, the common side effects are reviewed by experts who are oncology clinicians and clinical pharmacists 3. The completion percentage was added at the top of the survey screen so that patients can see their progress   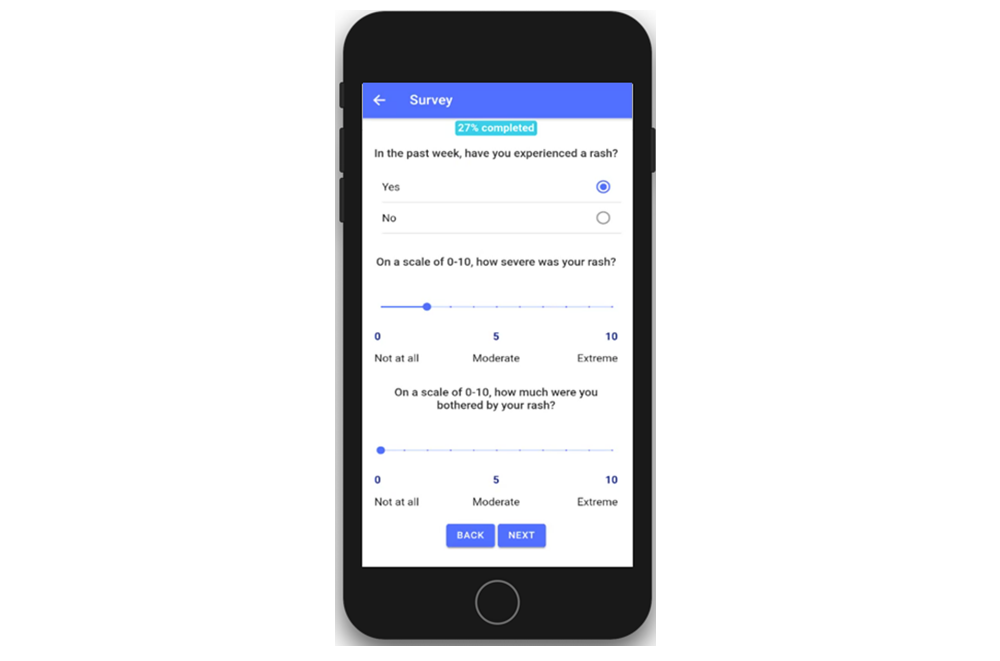   1. The “sort” and “search” functions were added to the web page 2. Drugs’ generic names and Anatomical Therapeutic Chemical classification codes were added as attributes 3. The “welcome” page was added to the app along with “Terms and conditions” and an introduction to SAMSON 4. Side effects images were added in the “side effects” tab of the app 5. The medication reminders and side effects surveys are grouped together on the home screen with better visual design   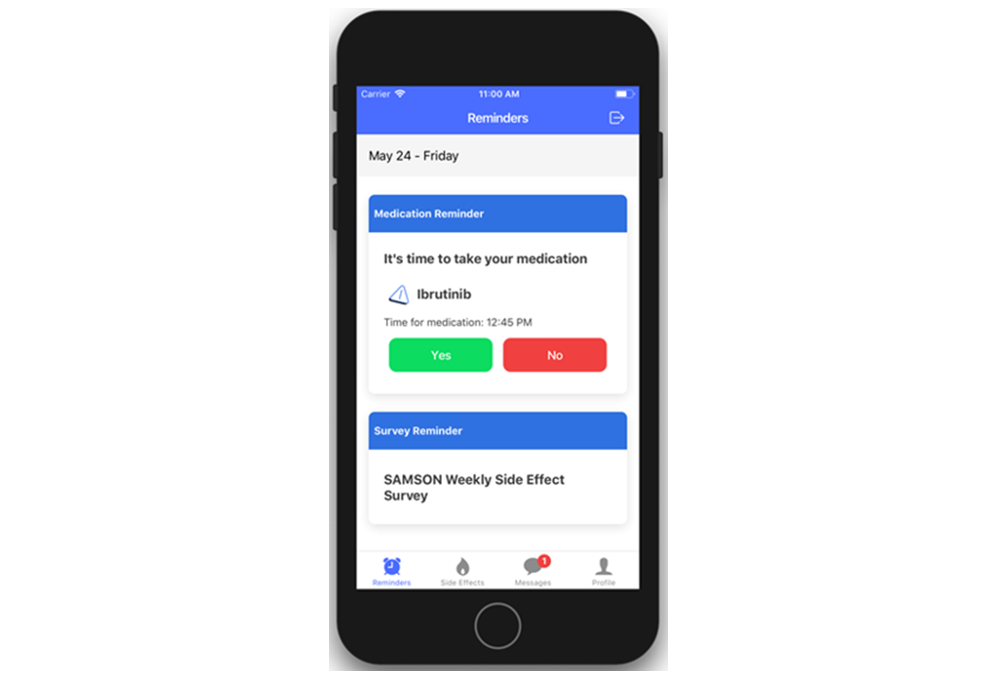 |
| --- |
