# Supplementary figures and images for: Co-Design, Development, and Evaluation of a Mobile Solution to Improve Medication Adherence in Cancer: Design Science Research Approach
Source: JMIR Cancer. 2024 Apr 3;10:e46979. doi: 10.2196/46979 (PMC11024750; doi:10.2196/46979)

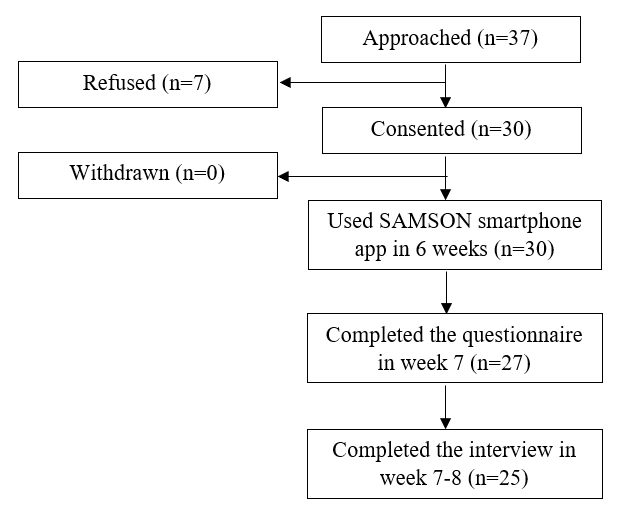


Appendix 5. CONSORT flowchart of participants completing the questionnaire and interview

Supplement: Multimedia Appendix 5 [file cancer_v10i1e46979_app5.doc]
