# Supplementary material for: Co-Design, Development, and Evaluation of a Mobile Solution to Improve Medication Adherence in Cancer: Design Science Research Approach
Source: JMIR Cancer. 2024 Apr 3;10:e46979. doi: 10.2196/46979 (PMC11024750; doi:10.2196/46979)
Supplement: Multimedia Appendix 7 [file cancer_v10i1e46979_app7.doc]

Appendix 7. Codebook

1. **SAMSON is a generally helpful app that can remind, support, and inform**
   1. App features often reliably help medication adherence
      1. Medication reminders remind patients to take their pills on time

- The reminder does its job
- The reminders can promote adherence in many different ways
- Reliable source of information
  - 1. The side effects surveys and management information improve patients' capability to better monitor and self-manage their symptoms
- Reasonable frequency of the survey
- Side-effect self-care advice is helpful
- Side-effect surveys can help patients better report their symptoms to clinicians when visiting
- Side-effects surveys are a good source of information for clinicians
- Side-effects surveys are simple
- Side-effects tab information is helpful
- Sub-questions are reasonable
- The survey functions well
- The survey help patients think about their adherence
- The survey helps patients think about their symptoms and track them
- The survey helps patients to recall and think about symptoms
- The survey's content is good
  - 1. Reinforcement messages can motivate adherence and increase the responsiveness of patients
- Content of reinforcement messages is good
- Messages tab is fine
- Reinforcement messages can make patients comply more with the use of the SAMSON app
- Reinforcement messages can motivate patients if they reflect the adherence rate correctly
- Reinforcement messages can remind patients to adhere to treatment
- Reinforcement messages are useful
- Reminder message comes as it means to be
- Some people are not concerned when reporting adherence rates inaccurately
- The message can motivate patients who start the treatment
- The way reinforcement messages were worded is reasonable
  - 1. Overall, the app is acceptable, usable, and useful
- App’s content is good
- Content of the reminder message is fine
- Don’t care about the content of the message
- Functionality – app functions well and could be improved
- Mostly acceptable app but occasionally stressful
- Presentation of the reminder message is simple and nice
- Profile tab can be helpful
- SAMSON app is acceptable
- Simple app is easy to use and carer support could help
- Visual design positively received
  1. Occasional users don't need the app, because they already established a good medication routine or different adherent strategies
     1. Patients who have been on treatment for a long time often established a medication-taking routine and don't need the app
- Occasional people don't need an app to adhere
- People who already established good adherence
  - 1. Some patients, who have been using different workable adherence strategies, weren't interested in the app

**2.** **Possible barriers encompass app glitches and users' technical inexperience**

2.1. App glitches need repair

- Patients take the drug but do not respond to the message, leading to incorrect adherence rate reflection
- The inaccurate adherence rate can make patients confuse
- The message didn't come with a tone
- The notification sits in the background
- The performance doesn't truly reflect patients' adherence

2.2. Some people are technical inexperienced and apprehensive

- Technical inexperienced people may feel reluctant to use the app
- Some subjective difficulties need support beyond the app

**3.** **Users’ desires related to SAMSON include content and feature refinement, customization, and connections to carers and healthcare professionals**

3.1. Content and feature refinement would increase engagement and support adherence

- Visual design can be more eye-catching
- Improved available features can enhance the adherence effect
- Some suggested new features to support adherence and safe medication

3.2. More customized content and features would support and encourage

- Some features can increase patients' autonomy
- Some features can make the app more personalized

3.3. Connections to carers and healthcare professionals could increase engagement

- Connections to carers could increase engagement
- Connections to healthcare professionals could increase engagement
